# Comparison and Interpretation of Taxonomical Structure of Bacterial Communities in Two Types of Lakes on Yun-Gui plateau of China

**DOI:** 10.1038/srep30616

**Published:** 2016-07-27

**Authors:** Maozhen Han, Yanhai Gong, Chunyu Zhou, Junqian Zhang, Zhi Wang, Kang Ning

**Affiliations:** 1 Key Laboratory of Molecular Biophysics of the Ministry of Education, College of Life Science and Technology, Huazhong University of Science and Technology, Wuhan, Hubei 430074, China; 2 Single-Cell Center, CAS Key Laboratory of Biofuels and Shandong Key Laboratory of Energy Genetics, Qingdao Institute of Bioenergy and Bioprocess Technology, Chinese Academy of Sciences, Qingdao, Shandong 266101, China; 3 State Key Laboratory of Freshwater Ecology and Biotechnology, Institute of Hydrobiology, Chinese Academy of Sciences, Wuhan, Hubei 430072, China; 4 Key Laboratory for Environment and Disaster Monitoring and Evaluation of Hubei, Institute of Geodesy and Geophysics, Chinese Academy of Sciences, Wuhan, Hubei 430077, China

## Abstract

Bacterial communities from freshwater lakes are shaped by various factors such as nutrients, pH value, temperature, etc. Their compositions and relative abundances would undergo changes to adapt the changing environments, and in turn could affect the environments of freshwater lakes. Analyses of the freshwater lake’s bacterial communities under different environments would be of pivotal importance to monitor the condition of waterbody. In this study, we have collected freshwater samples from two lakes on Yun-Gui plateau of China, Lake Dianchi and Lake Haixihai, and analyzed the bacterial community structures from these samples based on 16S rRNA sequencing. Results have shown that: Firstly, the bacterial community of these samples have very different taxonomical structures, not only between two lakes but also among the intra-groups for samples collected from Dianchi. Secondly, the differences between samples from two lakes are highly associated with the chemical-geographical properties of the two lakes. Thirdly, for samples of Dianchi and Haixihai, analytical results of physicochemical, taxonomical structure and relative abundance of community revealed that extreme physicochemical factors caused by human activities have strongly affected the bacterial ecosystem in Dianchi. These results have clearly indicated the importance of combining biological profiling and chemical-geographical properties for monitoring Chinese plateau freshwater bacterial ecosystem, which could provide clues for Chinese freshwater ecosystem remediation on plateau.

It is well-known that freshwater habitats, especially bacterial communities, play essential roles in global biogeochemical cycles. The taxonomical structure and relative abundance of bacterial community in freshwater would undergo changes to adapt the changing environments, and they would in turn affect the environments of freshwater lakes. As reported, the quality of freshwater has already been seriously affected by salinization, eutrophication and microbiological pollution^1^. Particularly, eutrophication, the biological response to the excess input of nutrients mainly introduced by human activities^2^ into a water body, can arise under natural conditions. For example, cyanobacteria blooms, which occurred as a result of the rapid growth of *Cyanobacteria*, especially from genus *Microcytis*^3^,^4^ with complex communities of photosynthetic and heterotrophic microorganisms, are often associated with the production of a series of different secondary metabolites produced by members of the genera *Anabaena, Microcystis, Planktothrix*^5^, *Aphanizomenon* and *Cylindrospermopsis*^6^. The physicochemical characters of freshwater, such as temperature and nutrition, would affect the taxonomical structure and relative abundance of species within the bacterial communities^7^ in a water body.

Therefore, it would be of pivotal importance to investigate the taxonomical structure of freshwater communities and the driving forces that could shape the bacterial communities in freshwater, which would provide clues for freshwater ecosystem remediation. Previous studies have showed nitrogen, phosphorus^8^ and temperature^9^,^10^ as main driving forces that could alter the taxonomical structure of bacterial community in a surface water body. However, considering geographical regions, previous researches mainly studied oceans, rivers or plain lakes^11^,^12^, the precise information of the bacterial community in plateau lakes is still deficient. Furthermore, the subjects of these studies were mainly focused on the taxonomical composition of sediment community^7^. Therefore, there is a lack of studies on bacterial communities in Chinese plateau freshwater lakes.

It is generally known that lakes could exist in two alternative stable states of oligotrophy (clear with abundant submerged macrophytes) and eutrophy (turbid with abundant algae). These two types of lakes have different geobiochemical characteristics^13^. Comparing the structure of bacterial communities of these lakes not only will help us to have a better understanding of the environmental factors’ driving mechanisms of bacterial communities in the freshwater lakes, but also could provide valuable information to protect and remediate these lakes. Hence, it is essential to explore the bacterial communities in Chinese plateau freshwater lakes.

In this work, we have chosen two Chinese plateau freshwater lakes, Dianchi (DC) and Haixihai (HXH), from where community samples were collected and compared. DC is the largest plateau lake in China, located on the Yun-Gui plateau, Southwestern of China. Due to the aggravation of human activities, many parts of the lake have been seriously deteriorated by eutrophication and toxic cyanobacteria bloom. HXH is a relatively non-polluted, macrophytic and oligotrophic freshwater lake, also located on the Yun-Gui plateau, about 300 kilometers from DC. These two lakes represent two types of lakes: DC is eutrophic, turbid with abundant algae; while HXH is oligotrophic, clear with abundant submerged plants. By comparing the structure of bacterial communities of these lakes, we could be able to better understand the mechanisms through which environmental factors could alter bacterial communities in the freshwater lakes, which may provide clues for eutrophic lake remediation.

16S rRNA sequences of environmental DNA were commonly used to study the bacteria composition and diversity of natural bacterial communities^7^, decoding the taxonomical structure of communities with fairly high resolution. More importantly, uncultured bacteria in the community could be assessed qualitatively and quantitatively. In this study, we have used Illumina MiSeq platform for sequencing the V4-V5 regions of the 16S rRNA gene to profile the bacterial community’s patterns of samples collected from DC and HXH.

Based on 16S rRNA sequencing and data analyses for bacterial communities in two plateau lakes, we seek answers for the following questions: (*i*) Are taxonomical structures of bacterial communities of DC different from those of HXH? (*ii*) How do environmental factors affect the structure of bacterial communities? What are key environmental factors that could differentiate samples? (*iii*) What species could serve as biomarkers to differentiate samples from two lakes or those from intra-groups of DC?

## Results and Discussions

### General statistical information of environmental variables and sequencing data

**Figure 1:**
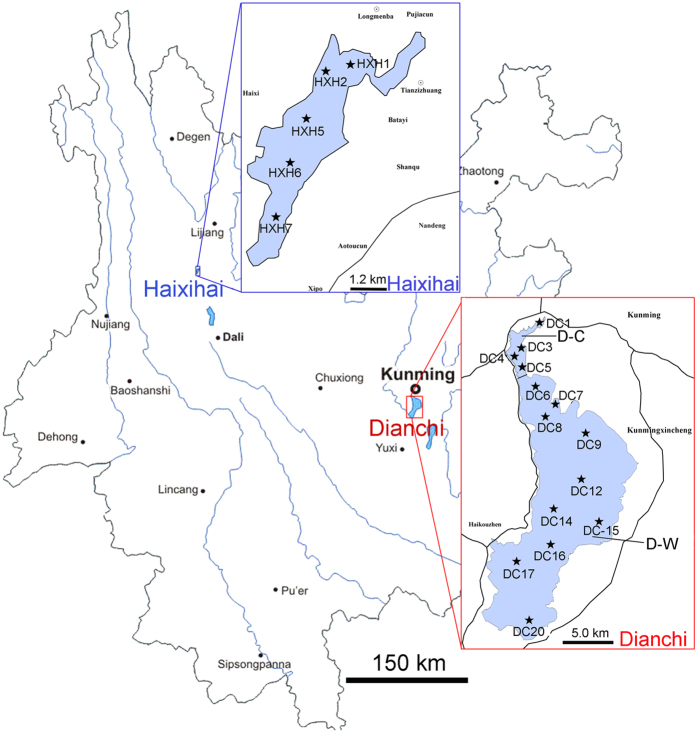
The geographical locations of DC and HXH and the sampling sites for this study. Red box indicates sampling location for DC samples, blue box indicates sampling location for HXH samples. The geographical locations were drawn using Adobe Photoshop and Adobe Illustrator.

Environment characterization: 19 samples (14 samples from DC, and 5 samples from HXH, each from a different site, Fig. 1) were collected and analyzed. These samples were collected at locations with different environmental characteristics.

Samples from DC and HXH have shown strong variations in dissolved oxygen (DO), total nitrogen (TN), ammonia nitrogen (NH_4_^+^-N), nitrate nitrogen (NO_3_^−^-N), nitrite nitrogen (NO_2_^−^-N), total phosphorus (TP), orthophosphate (PO_4_^3−^-P), suspended solids (SS), potassium permanganate index (COD_Mn_) and chlorophyll *a* (Chl-*a*) (*t*-test for per characterization, all *P* < 0.05, the value of each physicochemical character was shown in Supplementary Table S1). Phytoplankton biomass (proxied by Chl-*a*) of DC was higher than that of HXH. For intra-group samples from DC, TN (6.38–9.35 mg/L), TP (0.157–0.558 mg/L) and Chl-*a* (205.3–364.1 μg/L) in most of Caohai Lake (D-C) samples were all higher than what has been measured in Waihai Lake (D-W) (TN, 1.75–4.27 mg/L; TP, 0.096–0.205 mg/L; Chl-*a*, 57.2–148.6 μg/L), except DC7.

#### General statistics of sequencing results

For all of the 19 samples, 3,904,457 paired raw reads with read length of 301 bp were obtained. The number of reads per sample ranged from 222,894 to 428,888, with an average of 303,809 (Table 1). After trimming, screening, removing chimeras and singletons, 107,846 unique high quality reads per sample (2,047,735 reads in total for 19 samples) were obtained for downstream analysis.

#### Richness and diversity analyses based on Operational Taxonomic Units

**Figure 2:**
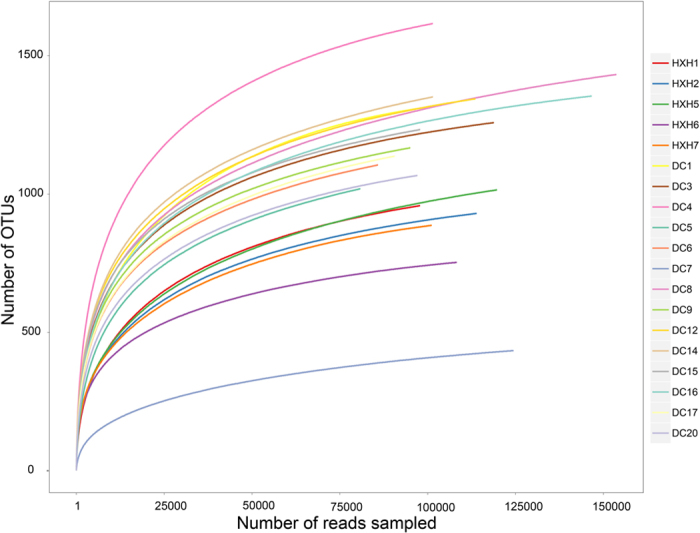
Rarefaction curves of OTUs for the bacterial communities’ samples from DC and HXH. The rarefaction curves of determined tags tend to approach the saturation plateau.

A total of 3,836 OTU were obtained from 19 samples. Samples from DC contain more OTUs than HXH except sample DC7 (Table 1), probably because community diversity of DC7 has been damaged by the extreme environment (with very high TN and COD_Mn_ values). The GOOD’s coverage of these samples was estimated to be between 99.72% and 99.91% (Table 1). The rarefaction curves based on the OTUs (Fig. 2) have shown that all the samples tend to approach the saturation plateau, indicating that these two freshwater lakes have around 1,000~2,000 bacterial species. Among DC samples, the ACE and Chao1 values at 97% similarity levels were higher than those in HXH samples (Table 1), except sample DC7, meaning that the bacterial richness of DC samples was higher than those in HXH samples (Table 1). In general, the diversity of HXH was lower than that of DC, indicating that the ecological environment of HXH might be more fragile.

### Comparison of the composition of bacterial communities.

Bacterial community composition in Lake Dianchi and Lake Haixihai: Based on the OTUs’ information of each sample (except DC7), we found out that the top 20 OTUs represented the accumulative relative abundance of 35.24% and 58.25% in DC and HXH communities, respectively; and they have shared 7 OTUs in common (Table 2). Further analyses revealed that 163 abundant OTUs (in top 200 OTUs, except DC7) had significant differences between samples from DC and those from HXH (Supplementary file 1). Phylogenetic relationship analysis and relative abundance analysis on the top 200 OTUs (except DC7) have shown that these OTUs belong to the few most abundant phyla and their relative abundances varied between DC and HXH (Supplementary Figure S1).

Further analysis at phylum level showed that an average of 23.86 and 21.8 different phyla were identified for samples from DC and HXH. Dissimilarity tests based on the Bray-Curtis distance revealed that the community differences in phylum level between DC and HXH were significant (PERMANOVA, *F* = 8.354, *P* = 0.0004). Analyzed results revealed that the significant difference between DC and HXH could be owed to the existence of *Cyanobacteria* (Kruskal-Wallis test, except DC7, *P* = 0.003), *Planctomycetes* (Kruskal-Wallis test, except DC7, *P* = 0.002), *Chlorobi* (Kruskal-Wallis test, except DC7, *P* = 0.0008) and *Verrucomicrobia* (Kruskal-Wallis test, except DC7, *P* = 0.017).

**Figure 3:**
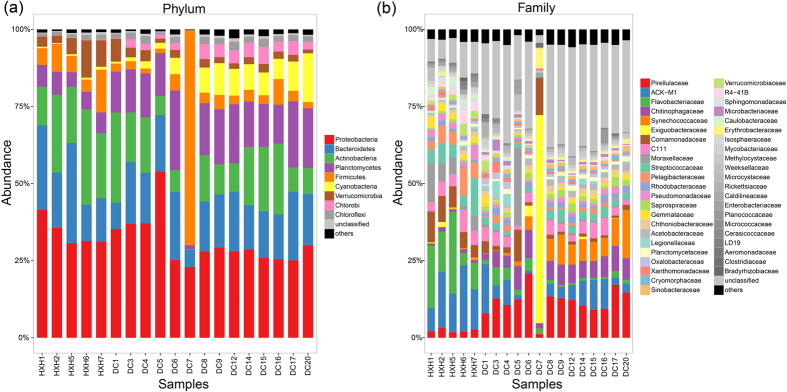
Taxonomical structure and relative abundance of each sample (a) at phylum level and (b) at family level. At specific level, “others” represent those accounting for < 1% of the total OTUs in each sample and are shown in black at the top of each bar.

More specifically, some taxa were more abundant in DC than in HXH at different taxonomical level. Firstly, as algal blooms related to eutrophication world-wide^14^, *Cyanobacteria* was characterized as the main causative organism^15^ (D-C, 0.65–2.33%, D-W, 5.31–15.83%). Secondly, in our study, *Synechococcaceae* (D-C, 0.12–1.52%, D-W, 4.38–15.67%) appeared as the main clade of *Cyanobacteria* (Fig. 3(b)) and dominated in DC samples compared to HXH. Thirdly, the *Planctomycetales* (0.35–1.35%) community amplified with *Pirellulales* (1.76–3.21%) can also be observed in large proportion in DC. Finally, the relative abundance of *Saprospirae* (a class of *Bacteroidetes*) had significant difference between DC and HXH (*t*-test, *P* < 0.05), which might be supported by a previous study, showing that the abundance of *Bacteriodetes* was associated with algae blooms^16^. The unclassified bacteria also took up a great proportion in samples from DC, indicating a much more complex taxonomical structure for samples in DC.

In contrast to samples of DC, *Flavobacterium* (a genus of *Bacteroidetes*, Fig. 3(b)) was the most abundant bacteria phylum in samples from HXH. In more details, HXH1-HXH5: 12.88–26.39%, HXH6 and HXH7: 4% and 8.68%. These value were small because of there were more than 49.9% OTU belonging to unclassified clades identified as the predominate group in the HXH6 and HXH7. Similarity phenomenon was found in various environments, such as soil and fresh water^17^. Studies showed that *Flavobacterium*, a heterotrophic bacteria that preferring an aerobic lifestyle^18^, was found to be abundant in freshwater and played a part in absorption and degradation of organic matter^19^. Moreover, *Comamonadaceae* (a family of *Betaproteobacteria*) and *Verrucomicrobia*, which have been discussed in a previous study showing that these taxa appear mostly in a specific degree of salinity^20^, account for a very large proportion of HXH (6.92% and 1.65%), but only a small percentage in DC (2.12% and 0.48%).

Although many environmental factors varied between DC and HXH, some dominant bacteria in samples from the two different lakes were similar. The main bacteria commonly existed in most freshwater lakes include *Alphaproteobacteria* (Table 2), which was abundant in both DC (7.30–19.86%) and HXH (12.47–18.37%). This indicated that *Alphaproteobacteria* was capable of living in both low nutrient environment and complex organic surroundings. Although the relative abundance of *Alphaproteobacteria* were moderate in many of the samples (Supplementary Figure S2), many studies have shown that they were ubiquitous and important for nitrogen cycle^21^. Additionally, *Actinobacteria,* especially *ACK-M1* (a family of *Actinobacteria*, Fig. 3(b)), dominant in both DC and HXH (but there is no statistical difference between DC and HXH for *Actinobacteria*; Kruskal-Wallis test, except DC7, *P* = 0.144). Studies have shown that *Actinobacteria* dominated in all kinds of circumstances including soil and aquatic system^21^. The role of protistan predation may explain why they were common to the plankton^22^. Furthermore, *Actinobacteria* showed higher level of diversity in DC (Fig. 3(a)). It was rational to deduce that some specific bacteria of *Actinobacteria* could serve as saprophytic microbes that prefers eutrophic conditions^23^.

**Figure 4:**
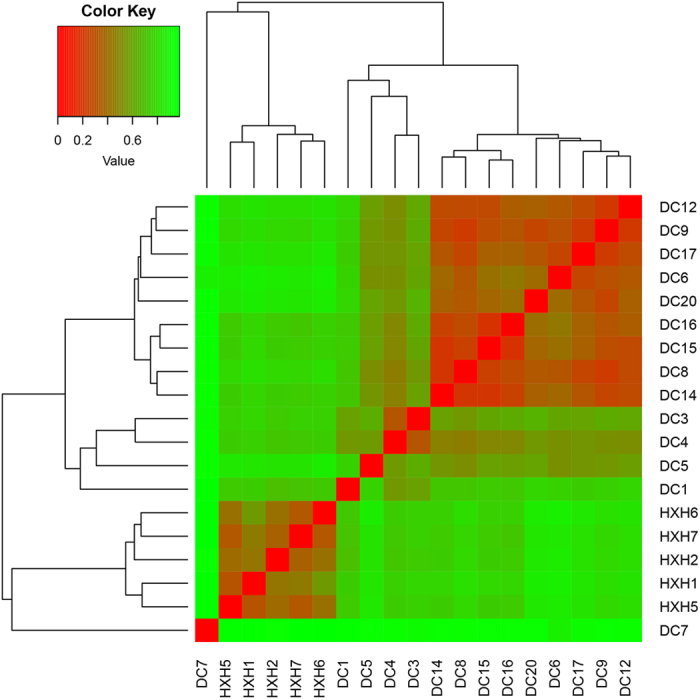
Heatmap representing the differences among samples based on Bray-Crutis distance measures. Bray-Curtis distances were calculated based on the formula: 
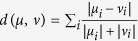
. Computes the distance between μ and ν sample. *i* represents the OTU *i* in each sample. Based on the Bray-Crutis distance, a matrix was created among all samples and then for heatmap analysis.

**Figure 5:**
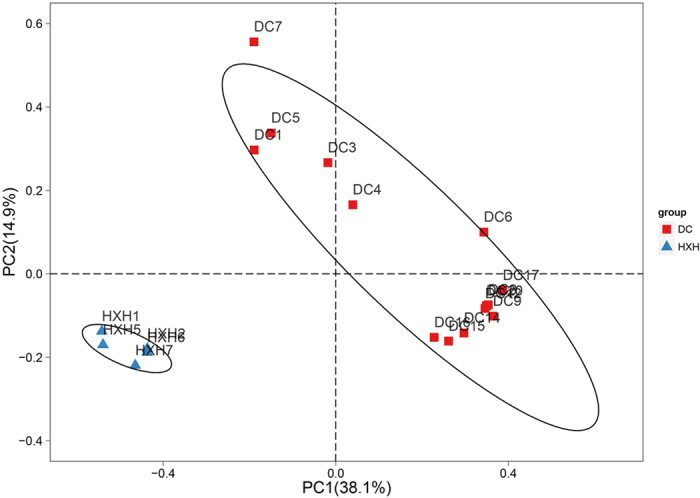
PCoA of the dissimilarities among bacterial community taxonomical structures using thetaYC distances. ThetaYC (
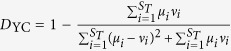
) measures the dissimilarity between the structures of two communities, where S_T_ is the total number of OTUs in communities μ and ν, μ_i_ and ν_i_ is the relative abundance of OTU *i* in community μ and ν, respectively. A matrix of pairwise thetaYC-based distances among all samples was calculated for PCoA analysis.

Furthermore, in order to examine the clustering patterns for samples from DC and HXH, a hierarchical clustering heatmap was generated based on all bacterial communities across all selected samples. The analyses have shown that DC7 was very distinct from other samples and samples of HXH were also different from samples of DC. Among samples from DC, samples other than the 5 samples (DC1, 3, 4, 5, 7) were clustered (Fig. 4). Samples from DC and HXH could also be distinguished by PCoA analysis (ANOSIM, *P* < 0.001), but group DC showed larger variance in one gradient direction (Fig. 5). Therefore, samples measured in DC and HXH could be clearly divided into three distinct clusters according to environmental factors: Cluster 1: samples in DC1, 3, 4, 5; Cluster 2: samples in DC6, 8, 9, 12, 14, 15, 16, 17, 20; Cluster 3: samples in HXH1, 2, 5, 6, 7. Additionally, the composition result, heatmap and PCoA result all revealed that DC7 was an exceptional sample. Therefore, comprehensive analytical results for DC7 would be detailed in a separate subsection.

### Relationship between environmental variables and bacterial communities

**Figure 6:**
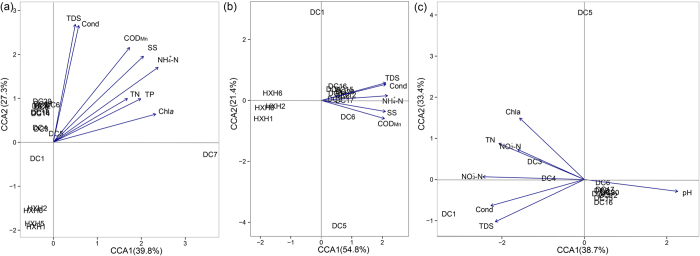
CCA plot showing the separation power of statistically significant environmental factors for differentiating samples. (**a**) Samples from DC and HXH; (**b**) Samples from DC and HXH (except DC7); (**c**) Samples from DC (except DC7).

Of the measured variables, we employed envfit^24^,^25^ as the analytical method in CCA package to identify a set of environmental factors that best explained the pattern of bacterial community clustering: TDS, Cond, TN, NH_4_^+^-N, NO_3_^−^-N, NO_2_^−^-N, TP, SS, pH, COD_Mn_ and Chl-*a* (Fig. 6). Results have shown that these factors: TN, TP, TDS, COD_Mn_, SS and T, were correlated with the differences between bacterial communities from DC and HXH. The CCA results for distinguishing samples from HXH and DC (Fig. 6(a)) have shown a distinctively isolated point (DC7 sample), which was mostly influenced by Chl-*a*, TN and TP. And this result was consistent with the previous work^8^, which demonstrated that concentrations of both nitrogen and phosphorus could influence the growth of freshwater cyanobacteria in an eutrophic environment. In contrast, TDS, Cond, NH_4_^+^-N, SS and COD_Mn_ were the apparent dominant factors determining the bacterial community in HXH and DC when we excluded the influence of DC7 (Fig. 6(b)), in keeping with the result of physicochemical analysis. Meanwhile, *Synechococcaceae* was observed as the dominating clade of Cyanobacteria in DC samples. And it has been proven that *Synechococcaceae* was the resource of nitrogen and could use chlorophyll and assortment of phycobilins to perform oxygenic photosynthesis^21^. *Planctomycetales* was observed in large proportion of DC where there was a higher degree of TN, which has played a part in anaerobic ammonium oxidation^26^. This was consistent with the observation that *Planctomycetales* might play an important role in the ammonia cycle in the DC aquatic system^27^. Therefore, it was noteworthy that TP, TN and Chl-*a* were key factors for setting sample of DC7 as an outlier, which may shed light on the reason of the appearance of *Exiguobacterium* dominating in DC7. When it came to DC only (excluding DC7, Fig. 6(c)), it was apparent that pH was the most influential factor. Compared to D-W, the samples in D-C (DC1, 3, 4 and 5) located dispersedly on the graphical representation of CCA, showing fewer environmental factors with impact to D-C but larger differences among samples. These results conclusively demonstrated that bacterial communities in DC was different from those in HXH, supporting the hypothesis that bacterial community structure was affected by chemical geological factors^7^. The testing result might be explained by external factors, one of which was that the urban sewage and part of industrial effluent flew directly into D-C. The result has further illustrated that different environment factors, especially nutrient variations, were playing essential parts in forming the bacterial community of three clustered samples (Cluster 1, Cluster 2 and Cluster 3).

Although both DC and HXH were plateau lakes, the different nutrient conditions have made their community structures vary greatly. For DC, about 45% of the area’s treated/untreated wastewaters flowed into the small sublake D-C (DC1, 3, 4, 5), which was only 3% of the total areas of DC. Therefore, samples from D-C have shown a higher degree of variation compared with other DC samples. Physicochemical analysis as shown in Supplementary Table S1 also suggested that three clusters (Cluster 1, Cluster 2 and Cluster 3, representing D-C, D-W and HXH, respectively) could be clearly separated according to their ecological conditions provided with different nutrient composition for the bacteria to reside and proliferate (Figs 4 and 5). More specifically, Cluster 1: DC1, 3, 4, 5 showed lower pH values than other samples in DC, but a higher concentration of TN contributing to eutrophication. Chl-*a*, which acts as the origin of carbon for bacterial and influences pH by photosynthesis, was higher in DC than HXH, indicating the eutrophication in DC as well. Previous study has shown bacterial community groups associated with a relatively higher level of planktonic algae biomass^28^. Therefore, Chl-*a* might be selected as an important index for the breakout of cyanobacteria blooms.

We further examined the pattern of bacterial community’s taxonomical structures for samples from these plateau lakes and those from a plain lake. In this work, Lake Taihu (eutrophic lake) was selected as the representative plain lake for comparing the differences in taxonomical structures between bacterial communities from plain lake and those from plateau lakes (DC and HXH). The taxonomical structures for bacterial communities in Lake Taihu were obtained from a recent study for this lake^29^. On OTU level, the number of bacterial OTUs (about 5,295) for samples from Lake Taihu were higher than those from DC and HXH, which might suggest that the bacterial communities of plain lake were more complicated than those of plateau lakes. We also compared the bacterial communities of Lake Taihu, DC and HXH on phylum level. The proportion of top 4 phyla from samples in Lake Taihu (97.2%) was higher than those from DC and HXH (about 77.93% on average). And more remarkable, *Actinobacteria* was presented in Lake Taihu as dominant group (60.7 ± 10.2%)^29^ and its proportion was 15.68 ± 8.09% in DC and HXH, whereas dominant bacterial phyla in DC and HXH was *Proteobacteria* (average: 31.68%, ranging from 22.97% to 53.92%). Hence, the bacterial composition for plateau lakes’ bacterial communities were different from those of the plain lake.

### Biomarker discovery and interpretation

To find out what species could be selected as biomarkers for such differences among DC (except DC7) and HXH, we chose relative abundance of top 200 OTUs, out of which 163 OTUs showed statistically significance (shown in Supplementary file 1) based on LEfSe method^23^, which may contribute to the differences. 163 OTUs could be classified into 10 phyla and 145 OTUs have statistically significant difference (*P* < 0.05) and 18 OTUs have highly significant variation (*P* < 0.01) between DC and HXH (shown in Supplementary file 1).

**Figure 7:**
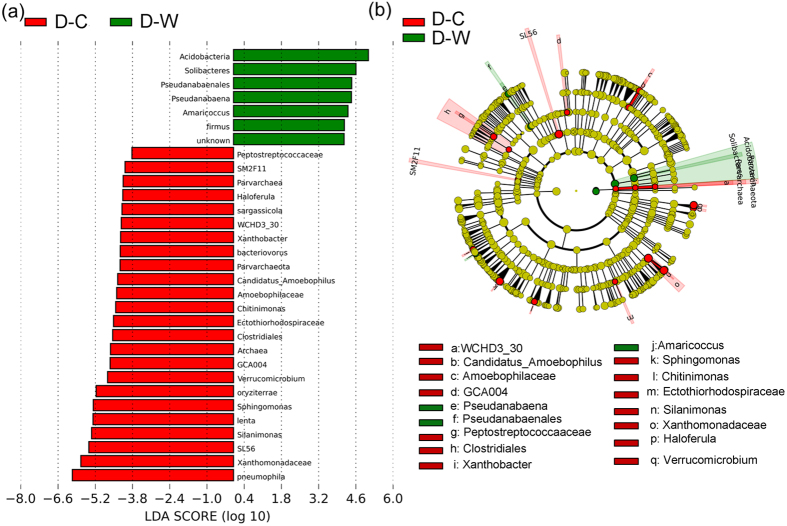
Comparison between bacterial community samples from D-W and D-C by indicator and cladogram. Samples divided into two groups according to the logarithmic LDA score, which were all higher than 3.8. (**a)** Sorted by degree of difference and (**b**) overlaid on a complete cladogram. Cladogram showed the bacterial distribution of two types of freshwater, and differences in abundance between them were presented as colors and circle’s diameters.

For intra-group of DC, although both D-C and D-W were of the same eutrophic environment in DC^30^, they were divided into two parts geographically by a man-made dike and biologically by the physicochemical conditions (Supplementary Table S1), which contributed to a result that discrepancy in bacterial community existed between samples in DC1, 3, 4 and 5 from other samples in DC (Figs 4 and 5). Except sample DC7, the community differences at phylum level between D-C samples and D-W samples were significant based on dissimilarity tests with Bray-Curtis distance (PERMANOVA, *F* = 6.584, *P* = 0.001). LEfSe was also applied to identify the difference among DC samples^23^. With the result from relative abundance of OTUs used as an input data, the 863 bacterial species could be divided into two distinct groups representing D-C and D-W, with a logarithmic LDA score (LEfSe uses LDA^31^ to estimate the effect size of each differentially abundant feature) no less than 3.8, demonstrating a high degree of difference in bacterial community between the two groups (Fig. 7(a)). Moreover, LEfSe highlighted several additional clades from class-level to genus-level showing differences between D-C and D-W. LEfSe reported 7 and 24 taxa to be more abundant with high logarithmic LDA score in D-W and D-C, respectively. In agreement with previous analyses, *Proteobacteria* were abundant in both D-W and D-C, but different clades in *Proteobacteria* possessed drastically different relative abundance: *Rhodobacterials* belonging to *Alphaproteobacteria* was the main clade of *proteobacteria* in D-W, while other clades like *Betaproteobacteria* and *Gammaproteobacteria* were more abundant in D-C. Other bacterial lineages enriched in D-C include *Amoebophilaceae* (a family within *Bacteroidetes*), GCA004 and SL56 within *Chloroflexi*, and *Verrucomicrobia*. Other biomarkers in D-W include *Firmus, Acidobacteria, Soilbactetres, Pseudanabaenales, Pseudanabaeba*, and *Amaricoccus*.

### Bacterial communities for an extreme case at DC7

DC7 was an extreme case of our analysis of bacterial community structures. As we explained above, DC7 had the most special physicochemical condition among DC samples with an extremely high value of TN, TP, SS and Chl-*a* (Supplementary Table S1), which was a typical phenomenon in eutrophication environment. The result of heatmap analysis, PCoA analysis and CCA analysis (Figs 4, 5, 6) all separated DC7 from other samples, demonstrating the discrepancy and uniqueness of it. The sampling site of DC7 was close to two inlets of the most polluted rivers (Daqing River and Hai River), where a great deal of untreated urban effluent might be discharged into D-W through the two rivers^32^. Largely due to extreme water physicochemical characters, the bacterial community of DC7 with less richness was also clearly different from other samples in DC. The bacteria dominated in DC, such as *Cyanobacteria* (0.08%) and *Planctomycetes* (1.22%), were a small quantity of bacteria community in DC7 as a result of extreme harsh environment. The dominant clade of DC7, *Firmicutes* showed a relatively high proportion (69.5%, Fig. 3(a)). Additionally, the saprophytic microbes like *Exiguobacterium*, was the dominant genus (67.5%, Fig. 3(b)), *Exiguobacterium* preferred to live in eutrophic conditions^23^. Previous study revealed that *Exiguobacterium* was an alkaliphile and widely distributed in saline environment^7^. Therefore, we speculated that untreated urban effluent that might be discharged from two rivers (Fig. 1) into D-W (DC7) and changed the environment of DC7 into a more alkaline character than other samples, leading to prosperous clades of *Firmicutes*, and especially *Exiguobacterium* genus under *Firmicutes*. Our study revealed the fact that extreme water physicochemical characters can deteriorate the native bacteria community and shape communities to be ones that could adopt the harsh environment.

## Conclusion

Analyses of freshwater lake’s bacterial community have great importance for environment monitoring: Bacterial communities in freshwater lakes would undergo changes in composition and relative abundance to adapt the changing environment, and they would in turn affect the freshwater lakes. Therefore, monitoring of bacterial communities and their surrounding physical-chemical-geographical properties would be important for the assessment of freshwater lake’s water quality.

In this study, we have collected 19 bacterial community samples from two plateau freshwater lakes DC and HXH of China, and analyzed the bacterial communities from these samples. Results have shown that the community structures were different for those from DC and HXH. And intra-group of DC, the samples from D-C and D-W were also different. We have found *Acidobacteria, Solibacteres, Pseudanabaenales*, which were more abundant in D-W, and *Pneumophila, Xanthomonadaceae* and *SL56* abundant in D-C, as prominent biomarkers that could represent different sets of samples among DC samples. Moreover, we have examined DC7 with extreme water physicochemical characters, and discovered that *Exiguobacterium* was dominant in DC7 as a saprophytic microbe preferring eutrophication.

The differences among these clusters of samples were associated with their physical-chemical-geographical environments. Canonical correspondence analyses have shown that the bacterial community of DC and HXH’s freshwater samples were shaped by multiple forces including TDS, Cond, TN, NH_4_^+^-N, NO_3_^−^-N, NO_2_^−^-N, TP, SS, pH, COD_Mn_ and Chl-*a* with different weights. And their concerted effects have shaped the bacterial communities as we have seen today.

These results have clearly indicated the importance of association of biological profiling and physical-chemical-geographical properties for examining Chinese plateau freshwater bacterial ecosystem: only in this way can we observe that genus *Synechococcaceae* has played an important role in the resource of nitrogen and oxygenic photosynthesis for samples in D-W, and *Pirellulales*, which was abundant in DC, played a part in anaerobic ammonium oxidation.

As for future works, since the temperature would affect the bacterial community composition dramatically, it would be valuable to collect more samples on a regular basis in different seasons, and more importantly only by collecting those samples could a predictive model be built that might be used to predict the environmental changes. Another direction of future research would be on collecting samples from more diverse lakes with more diverse physical-chemical-geographical properties, based on which a global picture could be drawn not only for more accurate predictive model, but also to establish a link between bacterial ecosystem for certain types of lakes or mid-sized ecosystems.

## Materials and Methods

### Study areas and sampling processes

DC is one of the most seriously polluted^33^, large (area 300 km^2^), shallow (average depth 4.2 m), freshwater lakes in China. The lake is located on the Yun-Gui plateau area of Southwestern China, near the southwestern outskirts of the capital of Yunnan Province, Kunming City. The water temperature ranges from 9.8 °C to 26.5 °C, with an annual average of about 16.0 °C, and the lake water is alkaline and eutrophic. The lake serves for many social and economic purposes, such as flood control, irrigation, transportation, water supply, fishery and tourism. In recent decades, DC has gradually lost its function of water supply due to eutrophication and cyanobacteria blooms, which has resulted in the shortage of drinking water in Kunming City. The lake is divided into two parts: Caohai Lake (D-C) and Waihai Lake (D-W), by a man-made dike. D-C, lies at the north of DC, occupying about 3% of total surface of DC, receiving about 45% of the area’s treated/untreated wastewaters flowed into the lake^30^. The average depth is around 2.5 m. Although D-C is seriously eutrophication, almost no algae bloom occurred in the past decade. This may be due to the serious chemical pollution and relatively short water retention time (about 3 months). D-W is the main water body of DC and accounts for 97% of the whole area of the lake. The average depth of D-W is about 4.3 m. D-W was also seriously eutrophication and heavily cyanobacteria bloom had occurred in warm seasons. The water retention time in this sub-lake was about 2 years. The pollutants in this sub-lake mainly come from nearby 22 flowing rivers. HXH is a relatively non-polluted, macrophytic freshwater lake. The area is about 2.6 km^2^ and average depth is about 10 m with its maximum depth of about 16 m. The lake is surrounded by mountains on three sides and the annual average water temperature is about 13 °C.

In order to investigate and compare the bacterial communities from various environmental conditions in plateau lakes, we chose an oligotrophic lake (HXH) and a eutrophic lake (DC) for investigation. A total of 19 sampling sites were chosen and samples collected during 25–29, July, 2014. The geographical distributions of these samples were shown in Figs 1 and 5 sites (HXH1, 2, 5, 6 and 7) were located in HXH, which were almost distributed offshore throughout the lake, except HXH2, which was located near the lakeshore. 14 sites (DC1, 3–9, 12, 14–17 and 20) were located in DC. Among these sites, 4 sites (DC1, 3, 4 and 5) belong to D-C, which were distributed in this sublake; 10 sites (DC6-9, 12, 14–17 and 20) were located in D-W, which were distributed in this sublake. At each sampling site, 1 L mixed water sample was collected with 0.5 m depth of the water column by a cylinder sampler. The water samples were stored with portable cooler with ice bag, and then carried to the laboratory for further microorganism and physicochemical characters analysis. The biological material (about 200 ml water and other parts were used for measuring the physicochemical character) of each station was size fractionated into individual samples by two kinds of filtration membranes: 20 μm tulle and 0.22 μm pore size filter membrane (Tianjin Jinteng Experiment Equipment Co., Ltd). Bacteria in the water samples were collected onto 0.22 μm pore size filter membrane. These filter membranes were sealed and stored at −20 °C until transported to the laboratory and then stored at −80 °C freezer before DNA extraction.

### Physicochemical analysis

Environmental variables were collected for all freshwater samples (Supplementary Table S1). Water temperature (T), pH, dissolved oxygen (DO), conductivity (Cond) and Total Dissolved Solids (TDS) were measured *in situ* by portable meter (YSI ProPlus, USA) at the sampling sites. The other environmental parameters contained total phosphorus (TP), orthophosphate (PO_4_^3−^-P), total nitrogen (TN), ammonia nitrogen (NH_4_^+^-N), nitrate nitrogen (NO_3_^−^-N), nitrite nitrogen (NO_2_^−^-N), suspended solids (SS), potassium permanganate index (COD_Mn_) and chlorophyll *a* (Chl-*a*) were assayed according the Standard Methods (APHA, 1998).

### DNA extraction

In the laboratory, the filter membrane was aseptically cut into quarters for DNA extraction. Unused quarters were restored at −80 °C freezer. Quarters used for extraction were isolated using the Water DNA Kit (Omega Bio-Tek, USA) as described by the manufacturer. Extracted DNA of each sample was stored at −20 °C.

### Library Preparation and Illumina MiSeq Sequencing

Next generation sequencing library preparation and Illumina MiSeq sequencing were conducted as follows: DNA samples were quantified using a Qubit^®^ 2.0 Fluorometer (Invitrogen, Carlsbad, CA) and DNA quality was checked on a 0.8% agarose gel. 5–50 ng metagenomic DNA was used to generate amplicons that cover small subunit ribosomal (16S rRNA) V4-V5 hypervariable regions of bacteria and archaea for each individual sample. The forward primer contained the sequence “GTGYCAGCMGCCGCGGTAA” and reverse primer contained the sequence “CTTGTGCGGKCCCCCGYCAATTC”. Sequencing library was constructed using a MetaVx^TM^ Library Preparation kit (GENEWIZ, Inc., South Plainfield, NJ, USA). Indexed adapters were added to the ends of 16S rDNA amplicons by limited cycle PCR.

DNA libraries were validated using an Agilent 2100 Bioanalyzer (Agilent Technologies, Palo Alto, CA, USA), and quantified by Qubit^®^ 2.0 and real time PCR (Applied Biosystems, Carlsbad, CA, USA). DNA libraries were multiplexed and loaded on an Illumina MiSeq sequencer according to manufacturer’s instructions (Illumina, San Diego, CA, USA). Sequencing was performed using a 2 × 300 paired-end (PE) configuration; image analysis and base calling were conducted by the MiSeq Control Software (MCS) on the Illumina MiSeq sequencer.

### Raw sequence process

The raw sequences of bacterial 16S rRNA gene amplicons were processed by MOTHUR^34^ version 1.33.3. To minimize the effects of random sequencing errors and avoid overestimates of phylogenetic relationship diversity^35^, the paired reads were processed by removing adapters, splicing using “make.contigs” command with default settings, and removing the reads containing ambiguous base call (N), longer than 460 bp and shorter than 360 bp. We also performed the “chimera.uchime” script by MOTHUR^34^ to check putative chimeras and removed the chimeras with “remove.seqs” command. Putative contaminants (comparing candidate sequences against the Greengenes database^36^ August 2013 release) were removed from our datasets. Singletons were also removed. The MiSeq sequencing data for 19 freshwater lakes’ samples were deposited to NCBI SRA database with Bioproject number PRJNA299273.

### Operational Taxonomical Units (OTU) assignment and taxonomy classification

The high-quality reads that passed MOTHUR quality control process were aligned using default parameters against the Greengenes database by MOTHUR^34^. Calculation of coverage percentage (GOOD’s coverage)^37^. Community richness diversity was calculated by estimating the numbers of OTUs based on the ACE^38^ and Chao1^39^ values at the 97% similarity levels. Community diversity index: Shannon index (Shannon)^40^ and Simpson index (Simpson)^41^ were also conducted. Rarefaction analysis were also performed using MOTHUR^34^, based on which representative sequences for shared OTUs, as defined by 97% similarity, were obtained. Relative abundances of the bacterial taxa at phylum, family and genus level were calculated and compared.

### Comparison of bacterial community structures

To probe into the causes for the differences among the bacterial communities, we compared the community structure at different taxonomical levels and analyzed the phylogenetic relationships of the top 200 OTUs qualitatively and quantitatively. A heatmap of Bray-Curtis distance^42^ from different samples was generated using the R package gplots. Pairwise ecological distances were also calculated for all samples using the thetaYC^43^ distance metric by MOTHUR^34^, which takes both community membership and relative abundance into account. These distances were then visualized by principal co-ordinate analysis (PCoA)^44^. Analysis of similarity (ANOSIM), which is a nonparametric test of differences between 2 or more groups based on any distance measure^45^, was used to statistically test the variation of bacterial community composition across sites. The significant values were computed based on permutation of group membership with 1,000 iterations. Permutational multivariate analysis of variance (PERMANOVA)^46^ with Bray-Curtis dissimilarity was used for comparing the community structure of each dataset and sub-dataset.

### Relationship between environmental variables and bacterial communities

Canonical correspondence analysis (CCA), which was one of the best methods for direct gradient analysis in community ecology^47^, was used for revealing the correlations between bacterial communities and environmental factors. This procedure was conducted in R with the CCA function of Vegan package. The principle of choosing the significance of environmental factors was assessed with the “envfit” function^24^,^25^, which fitted environmental vectors onto ordination, determined r^2^ for environmental factors and defined the significance of each environmental variable (999 permutations) on all components conjointly with permutation procedure. Finally, we chose the environmental factors, which could show moderate correlation with both component-1 and component-2 in CCA analysis.

### Biomarker analysis

Linear discriminate analysis (LDA) effect size (LEfSe)^48^, which is an algorithm for high dimensional biomarker discovery between two or more biological conditions or classes, was utilized to find biomarker in metagenomic analysis. In this work, LEfSe pipeline was used for discovering metagenomic biomarker^48^. For inter-group analyses, DC group (except DC7) and HXH were classified as two classes with subclasses at OTU level, and for intra-group of DC (except DC7), two groups have been formed for comparison: D-C (DC1, 3, 4, 5) and D-W group (DC6–9, 12, 14–17, and 20). Alpha value for the factorial Kruskal-Wallis test^49^ among classes and the P-value of pairwise Wilcoxon test between subclasses were all smaller than 0.05. Threshold on the logarithmic LDA score for discriminative features was set to be 2.0^50^.

## Additional Information

**How to cite this article**: Han, M. *et al*. Comparison and Interpretation of Taxonomical Structure of Bacterial Communities in Two Types of Lakes on Yun-Gui plateau of China. *Sci. Rep.* **6**, 30616; doi: 10.1038/srep30616 (2016).

## Supplementary Material

Supplementary Information

Supplementary Information

## Figures and Tables

**Table 1:** Number of sequencing reads and estimates for diversity of each sample.

Sample	# of Raw reads	# of Processed reads	OTUs at 3% difference	Chao1	coverage	ACE	Simpson	Shannon
DC1	301062	99466	1313	1372	99.8%	1427	0.017	4.975
DC3	342776	118812	1258	1356	99.8%	1397	0.011	5.247
DC4	308328	101454	1616	1736	99.8%	1789	0.008	5.609
DC5	222894	80859	1019	1205	99.7%	1227	0.073	4.085
DC6	242808	85863	1105	1272	99.7%	1301	0.015	5.073
DC7	326472	124409	434	512	99.9%	550	0.467	1.623
DC8	428888	153687	1432	1626	99.8%	1672	0.014	5.204
DC9	274541	95055	1167	1328	99.8%	1343	0.018	5.089
DC12	316871	113583	1344	1470	99.8%	1510	0.012	5.356
DC14	293206	101477	1351	1547	99.7%	1564	0.014	5.246
DC15	295834	97860	1233	1413	99.8%	1407	0.015	5.189
DC16	407023	146659	1354	1470	99.9%	1513	0.017	5.005
DC17	256328	90603	1137	1301	99.7%	1342	0.018	5.073
DC20	262383	97063	1067	1194	99.8%	1229	0.033	4.744
HXH1	267063	97833	958	1080	99.8%	1119	0.049	4.172
HXH2	324733	113927	930	1032	99.8%	1062	0.027	4.453
HXH5	313294	119715	1015	1185	99.8%	1218	0.071	4.076
HXH6	305185	108268	753	819	99.9%	856	0.037	4.299
HXH7	282687	101142	887	973	99.8%	1019	0.031	4.411

**Table 2:** Top 20 OTUs and their relative proportions in bacterial communities’ sampled from HXH and DC, respectively.

No.	HXH			DC (Except DC7)		
		%	Taxonomy	No.	%	Taxonomy
1	OTU004	11.59	*Flavobacterium, gelidilacus*	OTU001	5.63	*Synechococcaceae, Synechococcus*
2	OTU002	6.82	*Actinomycetales, ACK-M1*	OTU007	3.06	*Pirellulales, Pirellulaceae*
3	OTU005	5.62	*Actinomycetales, ACK-M1*	OTU010	2.67	*Pirellulales, Pirellulaceae*
4	OTU008	5.39	*Rickettsiales, Pelagibacteraceae*	OTU009	2.45	*Chitinophagaceae, Sediminibacterium*
5	OTU006	4.77	*Streptococcaceae, Lactococcus*	OTU002	2.19	*Actinomycetales, ACK-M1*
6	OTU018	3.61	*Acidibacteriales, C111*	OTU011	1.92	*Acinetobacter, guillouiae*
7	OTU012	3.31	*Burkholderiales, Comamonadaceae*	OTU013	1.80	*Alphaproteobacteria, Rhizobiales*
8	OTU024	2.01	*Pedosphaerales, R4-41B*	OTU006	1.79	*Streptococcaceae, Lactococcus*
9	OTU011	1.91	*Acinetobacter, guillouiae*	OTU017	1.58	*Acidibacteriales, C111*
10	OTU019	1.77	*Pseudomonas, fragi*	OTU005	1.46	*Actinomycetales, ACK-M1*
11	OTU049	1.65	*Moraxellaceae, Perlucidibaca*	OTU016	1.32	*Acetobacteraceae, Roseomonas*
12	OTU051	1.43	*Moraxellaceae, Acinetobacter*	OTU023	1.29	*Chlorobi, OPB56*
13	OTU079	1.20	*Gemmatales, Gemmataceae*	OTU015	1.26	*Alphaproteobacteria, Rhizobiales*
14	OTU064	1.12	*Comamonadaceae, Limnobacter*	OTU021	1.23	*Actinobacteria, Actinomycetales*
15	OTU015	1.08	*Alphaproteobacteria, Rhizobiales*	OTU014	1.04	*Chthoniobacteraceae, Candidatus_Xiphinematobacter*
16	OTU036	1.07	*Streptococcaceae, Lactococcus*	OTU008	0.98	*Rickettsiales, Pelagibacteraceae*
17	OTU081	1.06	*Caldilineaceae, Caldilinea*	OTU028	0.96	*Chlorobi, OPB56*
18	OTU026	1.00	*Mycobacteriaceae, Mycobacterium*	OTU030	0.95	*Pirellulales, Pirellulaceae*
19	OTU014	0.99	*Chthoniobacteraceae, Candidatus_Xiphinematobacter*	OTU029	0.82	*Pirellulales, Pirellulaceae*
20	OTU101	0.82	*Bacteria, Bacteroidetes*	OTU032	0.82	*Saprospirales, Chitinophagaceae*
