# Supplementary material for: Comparison and Interpretation of Taxonomical Structure of Bacterial Communities in Two Types of Lakes on Yun-Gui plateau of China
Source: Sci Rep. 2016 Jul 27;6:30616. doi: 10.1038/srep30616 (PMC4962099; doi:10.1038/srep30616)
Supplement: Supplementary Information [file srep30616-s1.doc]

**Supplementary information**

**Comparison and Interpretation of Taxonomical** **Structure of** **Bacterial Communities in Two Types of Lakes on Yun-Gui** **plateau of China**

Maozhen Han^1, 2┼^, Yanhai Gong ^2┼^, Chunyu Zhou^1┼^, Junqian Zhang^3^, Zhi Wang^4^*, Kang Ning^1, 2^*

*^1^ Key Laboratory of Molecular Biophysics of the Ministry of Education, College of Life Science and Technology, Huazhong University of Science and Technology, Wuhan, Hubei 430074, China*

*^2^ Single-Cell Center, CAS Key Laboratory of Biofuels and Shandong Key Laboratory of Energy Genetics, Qingdao Institute of Bioenergy and Bioprocess Technology, Chinese Academy of Sciences, Qingdao, Shandong 266101, China*

*^3^ State Key Laboratory of Freshwater Ecology and Biotechnology, Institute of Hydrobiology, Chinese Academy of Sciences, Wuhan, Hubei 430072, China*

*^4^ Key Laboratory for Environment and Disaster Monitoring and Evaluation of Hubei, Institute of Geodesy and Geophysics, Chinese Academy of Sciences, Wuhan, Hubei 430077, China*

^┼^ These authors contributed equally to this work.

^*^ Corresponding author. E-mail: ningkang@hust.edu.cn (Kang Ning), zwang@whigg.ac.cn (Zhi Wang)

**Supplementary Tables and Figures Legends**

**Supplementary Table S1. Environmental variables for freshwater samples.** DC refers to samples from Lake Dianchi, while HXH refer to samples from Lake Haixihai.

**Supplementary Figure S1. Phylogenetic analysis and relative abundance analysis results of the top 200 OTUs in each sample.** 13 samples from DC (except DC7, 4 from D-C and 9 from D-W) and 5 samples from HXH have been compared. Biomarker analysis showed that 163 OTUs have statistical significance between DC and HXH as can be seen in the figure, among 163 OTUs, 44 OTUs can be classified at Genus level and 10 can be classified at Specie level (see **Supplementary file**). Out of the 163 biomarkers, 18 that could significantly (Wilcoxon test with p-value < 0.01) differentiate the two groups have been annotated in the figure with "***" annotations. "Legend" is for comparison of those samples (outer circles), while "Color ranges" refer to the leaves of the phylogenetic tree (inter circle).

**Supplementary Figure S2. Taxonomical structure and relative abundance analysis results of each sample (a) at Class level; (b) at Order level; (c) at Genus level; (d) at Species level.** For each specific level, “others” represent those accounting for < 1% of the total OTUs in each sample and are shown in black at the top of each bar.

**Supplementary Table S1**

| Sample | pH | DO  (mg/L) | T  (°C) | Cond  (mS/cm) | TDS  (mg/L) | TN  (mg/L) | NH_4_^+^-N  (mg/L) | NO_2_^-^-N  (mg/L) | NO_3_^-^-N  (mg/L) | TP  (mg/L) | PO_4_^3-^-P  (mg/L) | SS  (mg/L) | COD_Mn_  (O_2_,mg/L) | Chl-*a*  (μg/L) |
| --- | --- | --- | --- | --- | --- | --- | --- | --- | --- | --- | --- | --- | --- | --- |
| DC1 | 8.06 | 11.6 | 23.4 | 643 | 429 | 8.62 | 1.02 | 0.237 | 5.93 | 0.157 | 0.018 | 61 | 6 | 205.3 |
| DC3 | 8.7 | 10.6 | 24.7 | 558 | 364 | 9.35 | 0.92 | 0.49 | 2.96 | 0.558 | 0.022 | 99 | 19.76 | 364.1 |
| DC4 | 8.64 | 9.6 | 24.7 | 562 | 370.5 | 8.37 | 0.74 | 0.525 | 2.94 | 0.369 | 0.04 | 84 | 19.6 | 268.3 |
| DC5 | 9.2 | 8.62 | 25.1 | 512 | 331.5 | 6.38 | 1.02 | 0.205 | 1.21 | 0.325 | 0.026 | 114 | 20.72 | 291.8 |
| DC6 | 9.25 | 9 | 23.5 | 503 | 338 | 4.27 | 1.21 | 0.136 | 0.73 | 0.205 | 0.04 | 115 | 18.8 | 148.6 |
| DC7 | 9.31 | 9.34 | 25.1 | 523 | 338 | 9.67 | 2.34 | 0.087 | 0.53 | 0.558 | 0.016 | 176 | 27.28 | 519.2 |
| DC8 | 9.28 | 7.48 | 23.2 | 510 | 344.5 | 3.35 | 0.95 | 0.001 | 0.58 | 0.173 | 0.02 | 74 | 17.2 | 94.9 |
| DC9 | 9.51 | 8.78 | 23.3 | 516 | 344.5 | 3.09 | 1.07 | 0.072 | 0.3 | 0.149 | 0.004 | 74 | 12.88 | 87.6 |
| DC12 | 9.53 | 9.25 | 23.1 | 518 | 351 | 2.98 | 0.95 | 0.001 | 0.27 | 0.145 | 0.002 | 88 | 14 | 97 |
| DC14 | 9.7 | 9.52 | 24.5 | 523 | 344.5 | 2.61 | 0.92 | 0.006 | 0.3 | 0.12 | 0.01 | 71 | 16.24 | 57.2 |
| DC15 | 9.6 | 9.69 | 23.3 | 531 | 357.5 | 2.52 | 0.89 | 0.002 | 0.26 | 0.096 | 0.006 | 112 | 12.08 | 63.1 |
| DC16 | 9.63 | 10.4 | 23.3 | 532 | 357.5 | 2.46 | 1.02 | 0.087 | 0.28 | 0.153 | 0.066 | 76 | 12.72 | 80.3 |
| DC17 | 9.97 | 14.75 | 25.5 | 539 | 351 | 3.19 | 0.85 | 0.002 | 0.27 | 0.124 | 0.002 | 88 | 19.6 | 73.3 |
| DC20 | 9.72 | 12.73 | 24 | 538 | 357.5 | 1.75 | 0.87 | 0.001 | 0.26 | 0.124 | 0.024 | 65 | 17.04 | 67.7 |
| HXH1 | 9.11 | 10.92 | 23.5 | 162 | 108.55 | 0.37 | 0.07 | 0.001 | 0.06 | 0.01 | 0.018 | 2 | 1.84 | 2.4 |
| HXH2 | 9.14 | 10.49 | 23.7 | 168.6 | 112.45 | 0.15 | 0.08 | 0.001 | 0.04 | 0.016 | 0.008 | 2 | 1.68 | 2.5 |
| HXH5 | 9.02 | 8.7 | 23.3 | 183.5 | 123.5 | 0.13 | 0.08 | 0.001 | 0.02 | 0.012 | 0.01 | 2 | 2.16 | 3.5 |
| HXH6 | 9 | 8.6 | 23.2 | 184.4 | 124.15 | 0.17 | 0.05 | 0 | 0.03 | 0.012 | 0.006 | 2 | 1.2 | 3.1 |
| HXH7 | -- | -- | -- | -- | -- | -- | -- | -- | -- | -- | -- | -- | -- | -- |

Note：--，not detected.


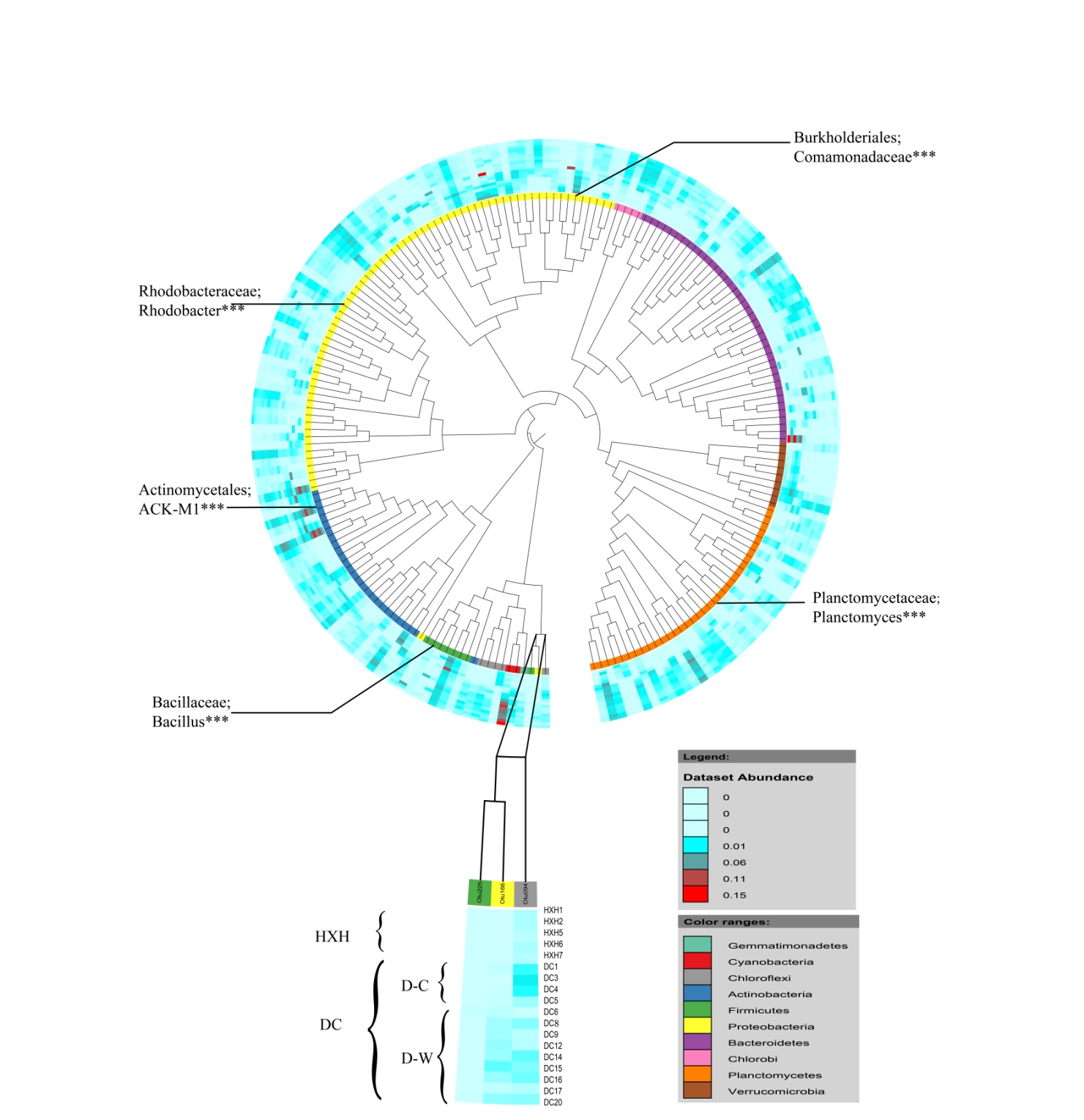


**Supplementary Figure S1. Phylogenetic analysis and relative abundance analysis results of the top 200 OTUs in each sample.** 13 samples from DC (except DC7, 4 from D-C and 9 from D-W) and 5 samples from HXH have been compared. Biomarker analysis showed that 163 OTUs have statistical significance between DC and HXH as can be seen in the figure, among 163 OTUs, 44 OTUs can be classified at Genus level and 10 can be classified at Specie level (see **Supplementary file**). Out of the 163 biomarkers, 18 that could significantly (Wilcoxon test with p-value < 0.01) differentiate the two groups. And out of the 18 significant biomarkers, 5 have been annotated in the figure with "***" annotations. "Legend" is for comparison of those samples (outer circles), while "Color ranges" refer to the leaves of the phylogenetic tree (inter circle).


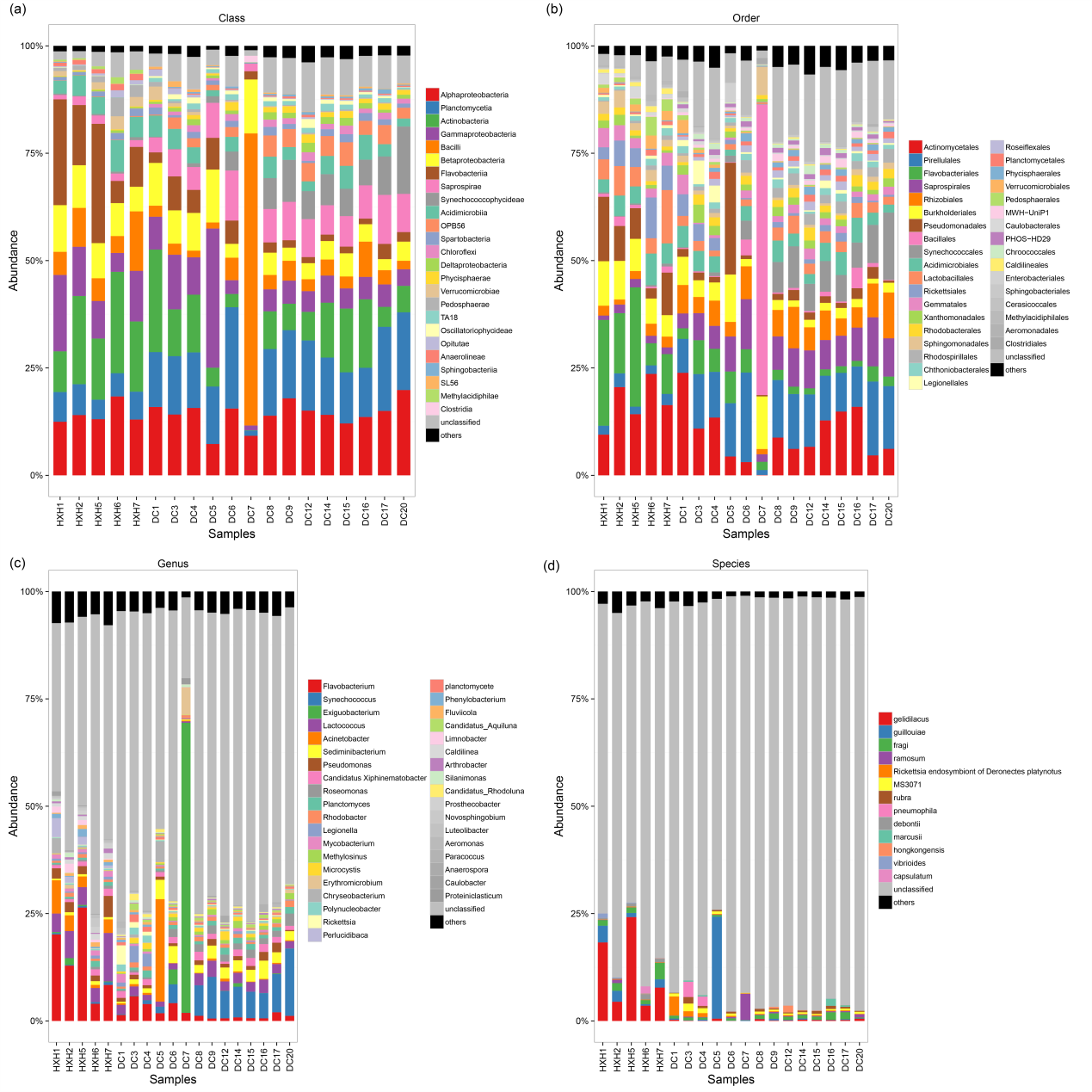


**Supplementary Figure S2. Taxonomical structure and relative abundance analysis results of each sample (a) at Class level; (b) at Order level; (c) at Genus level; (d) at Species level.** For each specific level, “others” represent those accounting for < 1% of the total OTUs in each sample and are shown in black at the top of each bar.
